# Error in Visual Abstract

**DOI:** 10.1001/jamanetworkopen.2025.56208

**Published:** 2026-01-09

In the Original Investigation titled “Comparison of Extended-Release Buprenorphine Doses for Treating High-Risk Opioid Use: A Randomized Clinical Trial”^1^ published online on December 17, 2025, there was an error in the Population section of the Visual Abstract. The number of women was 188, not 147. This article was corrected.^1^
